# Memories of the Future. Predictable and Unpredictable Information in Fractional Flipping a Biased Coin

**DOI:** 10.3390/e21080807

**Published:** 2019-08-18

**Authors:** Dimitri Volchenkov

**Affiliations:** Department of Mathematics and Statistics, Texas Tech University, 1108 Memorial Circle, Lubbock, TX 79409, USA; dimitri.volchenkov@ttu.edu

**Keywords:** fractional flipping a biased coin, probability entanglement, destructive interference of information

## Abstract

Some uncertainty about flipping a biased coin can be resolved from the sequence of coin sides shown already. We report the exact amounts of predictable and unpredictable information in flipping a biased coin. Fractional coin flipping does not reflect any physical process, being defined as a binomial power series of the transition matrix for “integer” flipping. Due to strong coupling between the tossing outcomes at different times, the side repeating probabilities assumed to be independent for “integer” flipping get entangled with one another for fractional flipping. The predictable and unpredictable information components vary smoothly with the fractional order parameter. The destructive interference between two incompatible hypotheses about the flipping outcome culminates in a fair coin, which stays fair also for fractional flipping.

*“A weaker man might be moved to re-examine his faith, if in nothing else at least in the law of probability.”*

Tom Stoppard, “Rosencrantz and Guildenstern Are Dead”, Act 1.

## 1. Introduction

The vanishing probability of winning in a long enough sequence of coin flips features in the opening scene of Tom Stoppard’s play “*Rosencrantz and Guildenstern Are Dead*”, where the protagonists are betting on coin flips. Rosencrantz, who bets on heads each time, has won 92 flips in a row, leading Guildenstern to suggest that they are within the range of supernatural forces. Furthermore, he was actually right, as the king had already sent for them [1].

Although coin-tossing experiments are ubiquitous in courses on elementary probability theory, and coin tossing is regarded as a prototypical random phenomenon of unpredictable outcome, the exact amounts of predictable and unpredictable information related to flipping a biased coin was not discussed in the literature. The discussion on whether the outcome of naturally tossed coins is truly random [2], or if it can be manipulated (and therefore predicted) [3,4] has been around perhaps for as long as coins existed. It is worth mentioning that tossing of a real coin obeys the physical laws and is inherently a deterministic process, with the outcome that, formally speaking, might be determined if the initial state of the coin is known [5].

All in all, the toss of a coin has been a method used to determine random outcomes for centuries [4]. The practice of flipping a coin was ubiquitous for taking decisions under uncertainty, as a chance outcome is often interpreted as the expression of divine will [1]. Individuals who are told by the coin toss to make an important change are reported much more likely to make a change and are happier six months later than those who were told by the coin to maintain the status quo in their lives [6].

If the coin is not fair, the outcome of future flipping can be either (i.) anticipated intuitively by observing the whole sequence of sides shown in the past in search for the possible patterns and repetitions, or (ii.) guessed instantly from the side just showed up. In our brain, the stored routines and patterns making up our experience are managed by the basal *ganglia*, and *insula*, highly sensitive to any change, takes care of our present awareness and might feature the guess on the coin toss outcome [7]. Trusting our gut, we unconsciously look for patterns in sequences of shown sides, *a priori* perceiving any coin as unfair.

In the present paper, we propose the information theoretic study of the most general models for “integer” and fractional flipping a biased coin. We show that these stochastic models are singular (along with many other well-known stochastic models), and therefore their parameters—the side repeating probabilities—cannot be inferred from assessing frequencies of shown sides (see Section 2 and Section 4). In Section 3, we demonstrate that some uncertainty about the coin flipping outcome can nevertheless be resolved from the presently shown side and the sequence of sides occurred in the past, so that the actual level of uncertainty attributed to flipping a biased coin can be lower than assessed by entropy. We suggest that the entropy function can therefore be decomposed into the predictable and unpredictable information components (Section 3). Interestingly, the efficacy of the side forecasting strategies (i.) and (ii.) mentioned above can be quantified by the distinct information theoretic quantities—the excess entropy and conditional mutual information, respectively (Section 3). The decomposition of entropy into the predictable and unpredictable information components is justified rigorously at the end of Section 3.

In Section 4, we introduce a backward-shift Markov chain transition matrix generalizing the standard “integer” coin flipping model for fractional order flipping. Namely, the *fractional order Markov chain* is defined as a convergent infinite binomial series in the “integer”-order transition matrix that assumes strong coupling between the chain states (coin tossing outcomes) at different times. The fractional backward shift transition operator *does not reflect any physical process*.

On the one hand, our fractional coin-tossing model is intrinsically similar to the *fractional random walks* introduced recently in [8,9,10,11,12] in the context of Markovian processes defined on networks. In contrast to the normal random walk where the walker can reach in one time-step only immediately connected nodes, the fractional random walker governed by a fractional Laplacian operator is allowed to reach any node in one time-step dynamically introducing a small-world property to the network. On the other hand, our fractional order Markov chain is closely related to the *Autoregressive Fractional Integral Moving Average* (ARFIMA) models [13,14,15], a fractional order signal processing technique generalizing the conventional integer order models—autoregressive integral moving average (ARIMA) and autoregressive moving average (ARMA) model [16]. In the context of time series analysis, the proposed fractional coin-flipping model resolves the fractional order time-backward outcomes (i.e., *memories* [17,18,19,20,21]) as the moving averages over all *future* states of the chain—that explains the title of our paper. We also show that the side repeating probabilities considered independent of each other in the standard, “integer” coin-tossing model appear to be entangled with one another as a result of strong coupling between the future states in fractional flipping. Finally, we study the evolution of the predictable and unpredictable information components of entropy in the model of fractional flipping a biased coin (Section 5). We conclude in the last section.

## 2. The Model of a Biased Coin

A biased coin prefers one side over another. If this preference is stationary, and the coin tosses are independent of each other, we describe coin flipping by a Markov chain defined by the stochastic transition matrix, viz.,

(1) $$T\left(p,q\right)=\left(\begin{array}{cc} p & 1-p\\ 1-q & q \end{array}\right),$$

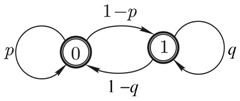

in which the states, ‘*heads*’ (“0”) or ‘*tails*’ (“1”), repeat themselves with the probabilities 0≤p≤1 and 0≤q≤1, respectively. The Markov chain Equation (1) generates the stationary sequences of states, viz., 0,0,0,⋯ when p=1, or 1,1,1,⋯ when q=1, or 0,1,0,1⋯ when q=p=0, but describes flipping a fair coin if $q=p=\frac{1}{2}$.

**Figure 1:**
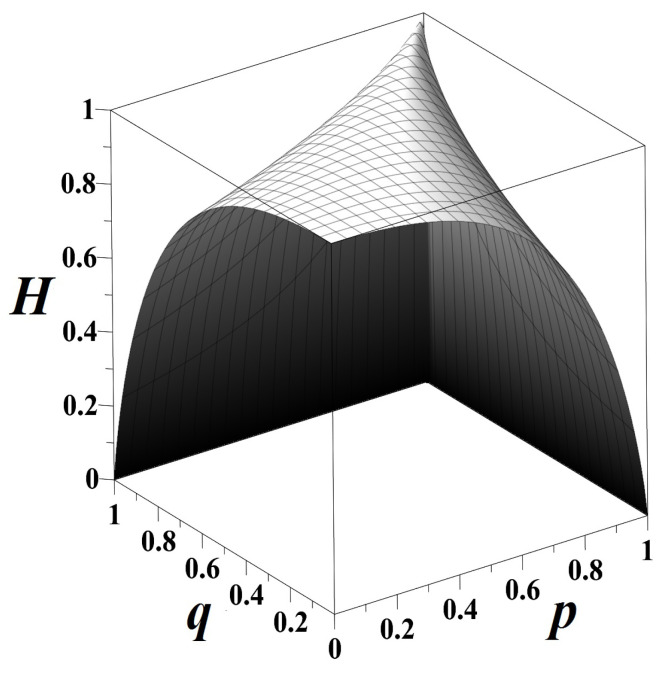
The value of entropy Equation (3) attains maximum (of 1 bit) for the symmetric chain, q=p, but is zero for the stationary sequences, p=1, or q=1.

For a symmetric chain, q=p, the relative frequencies (or densities) of ‘*head*’ and ‘*tail*’,

(2) $$\pi_{1}\left(p,q\right)=\frac{1-q}{2-p-q}\;\mathrm{and}\;\pi_{2}\left(p,q\right)=\frac{1-p}{2-p-q}\;\;,$$

are equal each other, $\pi_{1}\left(p,p\right)=\pi_{2}\left(p,p\right)=1/2,$ and therefore the entropy function, expressing the amount of uncertainty about the coin flip outcome, viz.,

(3) $$H\left(p,q\right)=-\sum_{k=1}^{2}\pi_{k}\left(p,q\right)\cdot \log_{2}\pi_{k}\left(p,q\right),$$

attains the maximum value, $H\left(p,p\right)=1$ bit, uniformly for all 0<p<1. On the contrary, flipping the coin when p=1 (or q=1) generates the stationary sequences of no uncertainty, $H\left(p,q\right)=0$ (see Figure 1). In Equation (3) and throughout the paper, we use the following conventions reasonable by a limit argument: $0\cdot \log0=\log0^{0}=\log1=0$. The information difference between the amounts of uncertainty on a smooth statistical manifold parametrized by the probabilities *p* and *q* is calculated using the *Fisher information matrix* (FIM) [22,23,24], viz.,

(4) $$g_{p,q}=\sum_{k=1}^{2}\pi_{k}\left(p,q\right)\cdot \frac{\partial }{\partial p}\log_{2}\pi_{k}\left(p,q\right)\cdot \frac{\partial }{\partial q}\log_{2}\pi_{k}\left(p,q\right).$$

However, since $H\left(p,p\right)=1$ bit, for 0<p=q<1, the FIM,

(5) $$g=\frac{1}{{\left(\ln2\right)}^{2}{\left(2-p-q\right)}^{2}}\;\left(\begin{array}{cc} \frac{1-q}{1-p} & -1\\ -1 & \frac{1-p}{1-q} \end{array}\right),$$

is degenerate (with eigenvalues $\lambda_{1}=0$, $\lambda_{2}=\left(p^{2}+q^{2}+2\left(1-p-q\right)\right)/\ln^{2}\left(2\right)\left(1-p\right)\left(1-q\right){\left(2-p-q\right)}^{2}$), and therefore the biased coin model Equation (1) is singular, along with many other stochastic models, such as Bayesian networks, neural networks, hidden Markov models, stochastic context-free grammars, and Boltzmann machines [25]. The singular FIM (4) assumes that the parameters of the model, *p* and *q*, cannot be inferred from assessing relative frequencies of sides in sequences generated by the Markov chain Equation (1).

## 3. Predictable and Unpredictable Information in the Model of Tossing a Biased Coin

Although coin tossing is traditionally regarded as a prototypical random experiment of unpredictable outcome, some amount of uncertainty in the model Equation (1) can be dispelled before tossing a coin. Namely, we can consider the entropy function Equation (3) as a sum of the *predictable* and *unpredictable* information components,

(6) $$H\left(p,q\right)\;\;=\;\;P\left(p,q\right)+U\left(p,q\right),$$

where the predictable part $P\left(p,q\right)$ estimates the amount of *apparent* uncertainty about the future flipping outcome that might be resolved from the sequence of sides shown already, and $U\left(p,q\right)$ estimates the amount of *true* uncertainty that cannot be inferred neither from the past, nor from present outcomes anyway. It is reasonable to assume that both functions, *P* and *U*, in Equation (6) should have the same form as the entropy function in Equation (3), viz.,

(7) $$P=-\sum_{k=1}^{2}\pi_{k}\cdot \log_{2}\varphi_{k},\;U=-\sum_{k=1}^{2}\pi_{k}\cdot \log_{2}\psi_{k},\;\varphi_{k}\psi_{k}=\pi_{k}.$$

Furthermore, as the more frequent the side, the higher the forecast accuracy, we assume that the partition function $\varphi_{k}$ featuring the predicting potential in already shown sequences for forecasting the side *k* is obviously proportional to the relative frequency of that side, $\varphi_{k}\propto \pi_{k}$. Denoting the relevant proportionality coefficient as $\sigma_{k}$ in $\varphi_{k}=\pi_{k}\sigma_{k}$, we obtain $\psi_{k}=\sigma_{k}^{-1}=\frac{\pi_{k}}{\varphi_{k}}$. Given the already shown sequence of coin sides ${\overset{\leftarrow }{S}}_{t}=S_{t-1},S_{t-2},S_{t-3},\ldots$, the average amount of uncertainty about the flipping outcome is assessed by the entropy rate [24] of the Markov chain Equation (1), viz

(8) $$H\left(S_{t}|{\overset{\leftarrow }{S}}_{t}\right)=H\left(S_{t}|S_{t-1}\right)=-\sum_{k=1}^{2}\pi_{k}\sum_{r=1}^{2}T_{kr}\log_{2}T_{kr},\;\mathrm{where}\;T=\left(\begin{array}{cc} p & 1-p\\ 1-q & q \end{array}\right),$$

and therefore, the *excess entropy* [25,26,27], quantifying the apparent uncertainty of the flipping outcome that can be resolved by discovering the repetition, rhythm, and patterns over the whole (infinite) sequence of sides shown in the past, ${\overset{\leftarrow }{S}}_{t}$, equals

(9) $$E\left(p,q\right)\equiv H\left(S_{t}\right)-H\left(S_{t}|S_{t-1}\right)=-\sum_{k=1}^{2}\pi_{k}\cdot \left(\log_{2}\pi_{k}-\sum_{r=1}^{2}T_{kr}\log_{2}T_{kr}\right).$$

**Figure 2:**
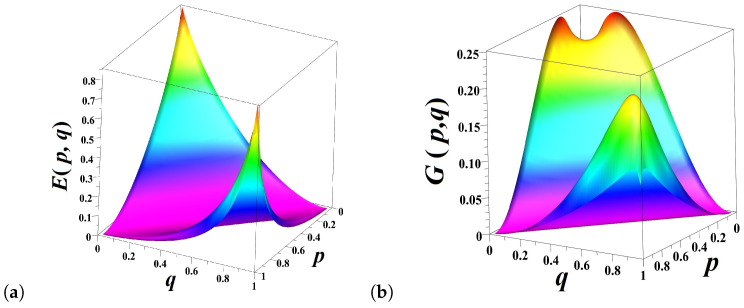
(**a**) $E\left(p,q\right)$, the apparent uncertainty of the flipping outcome that can be resolved by discovering possible patterns and repetitions in the infinite sequence of shown sides; (**b**) $G\left(p,q\right)$, the mutual information between the present state and the future state conditioned on the past measuring the efficacy of forecast of the coin toss outcome from the present state alone.

The excess entropy $E\left(p,q\right)$ attains the maximum value of 1 bit over the stationary sequences but equals zero for q=1−p (see Figure 2a).

Moreover, the next flipping outcome can be guessed from the present state alone, and the level of accuracy of such a guess can be assessed by the *mutual information* between the present state and the future state conditioned on the past state $I\left(S_{t};S_{t+1}|S_{t-1}\right)$ [25,28], viz.,

(10) $$\begin{array}{ccc} G\left(p,q\right) & \equiv & I\left(S_{t};S_{t+1}|S_{t-1}\right)\\ & = & H\left(S_{t+1}|S_{t-1}\right)-H\left(S_{t}|S_{t-1}\right)=\sum_{k=1}^{2}\pi_{k}\sum_{r=1}^{2}\left(T_{kr}\log_{2}T_{kr}-T_{kr}^{2}\log_{2}T_{kr}^{2}\right). \end{array}$$

The mutual information (10) is a component of the entropy rate (9) growing as p,q≳0 and p,q≲1. For q=1−p, the rise of *destructive interference* between two incompatible hypotheses on

(i) alternating the present side at the next tossing (if p,q>0), or
(ii) repeating the present side at the next tossing (when p,q<1)

causes the attenuation and cancellation of mutual information (10) (Figure 2b).

By summing (9) and (10), we obtain the amounts of predictable and unpredictable information, respectively:

(11) $$\begin{array}{ccccc} P\left(p,q\right) & = & E\left(p,q\right)+G\left(p,q\right) & = & H\left(S_{t}\right)-H\left(S_{t}|S_{t-1}\right)+I\left(S_{t};S_{t+1}|S_{t-1}\right),\\ U(p,q) & = & H(p,q)-P(p,q) & = & H\left(S_{t}|S_{t-1}\right)-I\left(S_{t};S_{t+1}|S_{t-1}\right)\\ & & & = & H\left(S_{t}|S_{t+1};S_{t-1}\right), \end{array}$$

where $H\left(S_{t}|S_{t+1};S_{t-1}\right)$ is the entropy of the present state conditional on the future and past states of the chain. The latter conditional entropy is naturally expressed via the entropy of the future state conditional on the present $H\left(\left.S_{t+1}\right|S_{t}\right)$, the entropy of the present state conditional on the past $H\left(\left.S_{t}\right|S_{t-1}\right)$, and the entropy of the future state conditional on the past $H\left(S_{t+1}|S_{t-1}\right)$ as following: $H\left(S_{t}|S_{t+1};S_{t-1}\right)=H\left(\left.S_{t+1}\right|S_{t}\right)+\left.H\left(\left.S_{t}\right|S_{t-1}\right)-H\left(\left.S_{t+1}\right|S_{t-1}\right)\right.$. The accuracy of the obtained information decomposition of entropy,

(12) $$H\left(p,q\right)\;\;=\;\;P\left(p,q\right)+U\left(p,q\right)\;\;=\;\;E\left(p,q\right)+G\left(p,q\right)+U\left(p,q\right),$$

is demonstrated immediately by the following computation involving the conditional entropies:

(13) $$\begin{array}{cc} H\left(S_{t}\right) & =H\left(S_{t}\right)-H\left(\left.S_{t+1}\right|S_{t}\right)+H\left(\left.S_{t+1}\right|S_{t}\right)\\ & =\left(H\left(S_{t}\right)-H\left(S_{t+1}|S_{t}\right)\right)+H\left(S_{t+1}|S_{t}\right)+\left\{H\left(S_{t}|S_{t-1}\right)-H\left(S_{t}|S_{t-1}\right)\right\}\\ & \;+\left\{H\left(S_{t+1}|S_{t-1}\right)-H\left(S_{t+1}|S_{t-1}\right)\right\}\\ & =\underset{E(p,q)}{\underset{︸}{\left(H\left(S_{t}\right)-H\left(S_{t+1}|S_{t}\right)\right)}}+\underset{G(p,q)}{\underset{︸}{\left(H\left(\left.S_{t+1}\right|S_{t-1}\right)-H\left(\left.S_{t}\right|S_{t-1}\right)\right)}}\\ & \;+\underset{U\left(p,q\right)}{\underset{︸}{\left(H\left(\left.S_{t+1}\right|S_{t}\right)+\left.H\left(\left.S_{t}\right|S_{t-1}\right)-H\left(\left.S_{t+1}\right|S_{t-1}\right)\right.\right)}}. \end{array}$$

**Figure 3:**
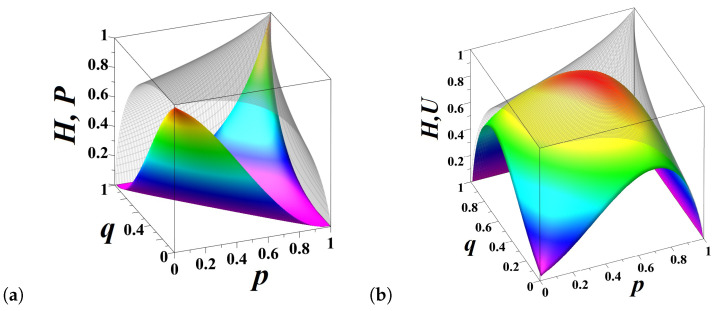
(**a**) The entropy $H\left(p,q\right)$ (transparent) and predictable information $P\left(p,q\right)$ (hue colored) in the model of a biased coin for the different values of *p* and *q*; (**b**) The entropy $H\left(p,q\right)$ (transparent) and unpredictable information $U\left(p,q\right)$ (hue colored) for the different values of *p* and *q*.

The predictable information component $P\left(p,q\right)$ amounts to $H\left(p,q\right)$ over the stationary sequences but disappears for q=1−p (Figure 3a). On the contrary, the share of unpredictable information $U\left(p,q\right)$ attains the maximum value $U\left(p,1-p\right)=H\left(p,1-p\right)$, for q=1−p (Figure 3b).

## 4. The Model of Fractional Flipping a Biased Coin

In our work, we define the model of fractional flipping a biased coin using the fractional differencing of non-integer order [29,30] for the discrete time stochastic processes [31,32,33]. The Grunwald-Letnikov fractional difference $\Delta_{\tau }^{\alpha }\equiv {\left(1-T\right)}^{\alpha }$ of order α with the unit step τ, and the time lag operator *T* is defined [18,29,30,34,35,36] by

(14) $$\Delta_{\tau }^{\alpha }x\left(t\right)\;\;\equiv \;\;{\left(1-T_{\tau }\right)}^{\alpha }x\left(t\right)=\sum_{m=0}^{\infty }{\left(-1\right)}^{m}\cdot \left(\begin{array}{c} \alpha \\ m \end{array}\right)\cdot x\left(t-m\cdot \tau \right)$$

where $T_{\tau }x\left(t\right)=x\left(t-\tau \right)$ is fixed τ-delay, and $\left(\begin{array}{c} \alpha \\ m \end{array}\right)$ is the binomial coefficient that can be written for integer or non-integer order α using the Gamma function, viz.,

(15) $$\left(\begin{array}{c} \alpha \\ m \end{array}\right)\equiv {\left(-1\right)}^{m-1}\frac{\alpha \cdot \Gamma (m-\alpha )}{\Gamma (1-\alpha )\Gamma (m+1)}.$$

It should be noted that for a Markov chain defined by Equation (1), the Grunwald-Letnikov fractional difference of a non-integer order 1−ε takes form of the following infinite series of binomial type, viz.,

(16) $${\left(1-T\right)}^{1-\varepsilon }=\sum_{k=0}^{\infty }\frac{\Gamma \left(k-1+\varepsilon \right)}{\Gamma \left(k+1\right)\Gamma \left(-1+\varepsilon \right)}T^{k}=1+\sum_{k=1}^{\infty }\frac{\Gamma \left(k-1+\varepsilon \right)}{\Gamma \left(k+1\right)\Gamma \left(-1+\varepsilon \right)}T^{k}\equiv 1-T_{1-\varepsilon }$$

that converges absolutely, for 0<ε<1. In Equation (16), we have used a formal structural similarity between the fractional order difference operator and the power series of binomial type in order to introduce a *fractional backward-shift transition operator* $T_{1-\varepsilon }$ for any fractional order 0<1−ε<1 as a convergent infinite power series of the transition matrix Equation (1), viz.,

(17) $$\begin{array}{ccccc} T_{1-\varepsilon }\;\left(p,q\right)\; & \equiv & -\sum_{k=1}^{\infty }\frac{\Gamma \left(k-1+\varepsilon \right)}{\Gamma \left(k+1\right)\Gamma \left(-1+\varepsilon \right)}T^{k}\left(p,q\right) & & \\ & = & \left(\begin{array}{cc} 1-\frac{1-p}{{\left(2-p-q\right)}^{\varepsilon }} & \frac{1-p}{{\left(2-p-q\right)}^{\varepsilon }}\\ \frac{1-q}{{\left(2-p-q\right)}^{\varepsilon }} & 1-\frac{1-q}{{\left(2-p-q\right)}^{\varepsilon }} \end{array}\right) & \equiv & \left(\begin{array}{cc} p_{\varepsilon } & 1-p_{\varepsilon }\\ 1-q_{\varepsilon } & q_{\varepsilon } \end{array}\right). \end{array}$$

The backward-shift fractional transition matrix defined by Equation (17) is a stochastic matrix preserving the structure of the initial Markov chain Equation (1), for any 0<1−ε<1. Since the power series of binomial type in Equation (17) is convergent and summable for any value 0<1−ε<1, we have also introduced in Equation (17) the *fractional probabilities, $p_{\varepsilon }$ and $q_{\varepsilon }$, as the corresponding elements of the fractional transition matrix.* The fractional transition operator Equation (17) describes fractional flipping a biased coin for 0<ε<1 as a *moving average* over the probabilities of *all future outcomes* of the Markov chain Equation (1) described by integer powers $T^{k}$, k=1,…,∞. The fractional Markov chain Equation (17) is also similar to the *fractional random walks* introduced recently in [8,9,10,11,12]. In these research efforts, the fractional Laplace operator describing anomalous transportation in connected networks and the fractional degree of a node are related to integer powers of the network adjacency matrix $A^{m}$ for m=1,…,∞ for which the element ${\left(A^{m}\right)}_{ij}$ is the total number of all possible trajectories connecting nodes *i* and *j* by paths of length *m*. The fractional characteristics of the graph not only incorporate information related to the number of nearest neighbors of a node, but also include information of all far away neighbors of the node in the network, allowing for long-range transitions between the nodes and featuring anomalous diffusion [10].

In the proposed fractional Markov chain Equation (17), the kernel function (which can be called *memory function* following [19,20,21,37]) establishes strong coupling between the outcome of fractional coin flipping for the fractional order parameter ε and the probabilities of all future outcomes of the “integer”-order Markov chain Equation (1). It is worth mentioning that the fractional transition probabilities in Equation (17) equal those in the “integer”-order flipping model Equation (1) as ε→0, viz.,

(18) $$\lim_{\varepsilon \to 0}p_{\varepsilon }=p,\;\lim_{\varepsilon \to 0}q_{\varepsilon }=q,$$

but coincide with the densities Equation (2) of the ‘*head*’ and ‘*tail*’ states, as ε→1, viz.,

(19) $$\lim_{\varepsilon \to 1}p_{\varepsilon }=\frac{1-q}{2-p-q}=\pi_{1},\;\mathrm{and}\;\lim_{\varepsilon \to 1}q_{\varepsilon }=\frac{1-p}{2-p-q}=\pi_{2}.$$

Thus, the minimal value of the fractional order parameter (ε=0) in the model Equation (17) may be attributed to the “integer”-order coin flipping when no information about the future flipping outcomes is available, i.e., the very moment of time when the present side of coin is revealed. Furthermore, the maximum value of the fractional order parameter (ε=1) corresponds to the maximum available information about all future coin-tossing outcomes. Averaging over all future states of the chain as ε=1 recovers the density of states Equation (19) of the Markov chain Equation (1) precisely as expected.

**Figure 4:**
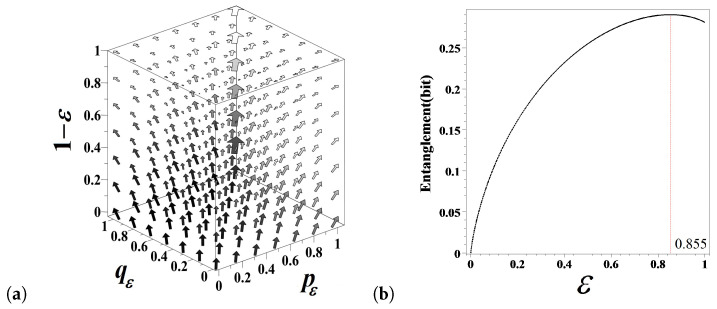
(**a**) The $\left(p_{\varepsilon },q_{\varepsilon }\right)$—flow of the model Equation (17) over fractional order 1−ε; (**b**) The entanglement between the probabilities $p_{\varepsilon }$ and $q_{\varepsilon }$ defined by Equation (20) attains the maximum value at ε=0.855.

The transformation Equation (17) defines the $\left(p_{\varepsilon },q_{\varepsilon }\right)$—flow of fractional probabilities over the fractional order parameter ε as shown in Figure 4a. In fractional flipping, 0<ε≤1, the state repetition probabilities $p_{\varepsilon }$ and $q_{\varepsilon }$ get entangled with one another due to the normalization factor ${\left(2-p-q\right)}^{-\varepsilon }$ in Equation (17). For the integer order coin flipping model ε=0, the state repetition probabilities $p_{0}$ and $q_{0}$ are independent of each other (as shown by flow arrows on to top face of the cube in Figure 4a) but they are linearly dependent, ${\pi_{1}=p}_{1}=1-q_{1}=1-\pi_{2}$, as ε=1 (see the bottom face of the cube in Figure 4a).

The degree of entanglement as a function of the fractional order parameter ε can be assessed by the expected divergence between the fractional model probabilities, $p_{\varepsilon }$ and $q_{\varepsilon }$, in the models Equation (1) and Equation (17), viz.,

(20) $$\begin{array}{ccccc} \mathrm{Ent}\left(\varepsilon \right) & = & {\int \int \;}_{0}^{1}\;dpdq\left(\pi_{1}\log_{2}\frac{p}{p_{\varepsilon }}+\pi_{2}\log_{2}\frac{q}{q_{\varepsilon }}\right) & = & \;2{\int \int \;}_{0}^{1}dpdq\left(\pi_{1}\log_{2}\frac{p}{p_{\varepsilon }}\right)\\ & & & = & 2\;{\int \int \;}_{0}^{1}dpdq\left(\pi_{2}\log_{2}\frac{q}{q_{\varepsilon }}\right). \end{array}$$

The integrand in Equation (20) turns to zero when the probabilities are independent of one another (as ε=0) but equals the doubled *Kullback–Leibler divergence* (relative entropy) [24] between *p* and $\pi_{1}$ (*q* and $\pi_{2}$) as ε=1 (due to the obvious p↔q symmetry of expressions). The degree of probability entanglement defined by Equation (20) attains the maximum value at ε=0.855 (Figure 4b).

Since the vector of ‘*head*’ and ‘*tail*’ densities Equation (2) is an eigenvector for all integer powers $T^{k}$, it is also an eigenvector for the fractional transition operator $T_{\varepsilon }\;\left(p,q\right)$, for any value of the fractional order parameter ε. Therefore, the fractional dynamics of transition probabilities does not change the densities of states in the Markov chain, so that the entropy function Equation (3) is an invariant of fractional dynamics in the model Equation (17) (Figure 4a). The Fisher information matrix Equation (4) is redefined for the probabilities $p_{\varepsilon },q_{\varepsilon }$, viz.,

(21) $$g_{p_{\varepsilon },q_{\varepsilon }}=\sum_{k=1}^{2}\pi_{k}\cdot \frac{\partial }{\partial p_{\varepsilon }}\log_{2}\pi_{k}\cdot \frac{\partial \;}{\partial q_{\varepsilon }}\log_{2}\pi_{k},$$

which is also degenerate because the symmetry p↔q is preserved in all the expressions for all values 0<ε<1. The nontrivial eigenvalue of the FIM Equation (21) turns to zero as well, for the stationary sequences with p=q=1. The fractional flipping a biased coin model is singular, as well as the integer time flipping model Equation (1).

## 5. Evolution of Predictable and Unpredictable Information Components over the Fractional Order Parameter

**Figure 5:**
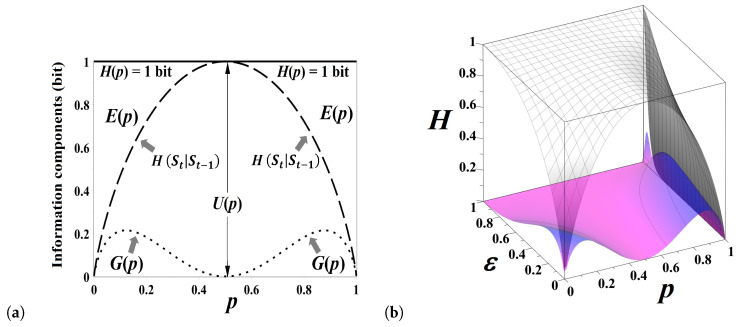
(**a**) The decomposition of entropy into information components for a symmetric unfair coin, q=p, for “integer”-order coin flipping. The symmetric coin is fair when p=1/2: the amount of uncertainty of the fair coin tossing cannot be reduced anyway, as the amount of unpredictable information equals U(1/2)=H(p)=1 bit; (**b**) The information components for fractional order flipping a biased coin. The decomposition of entropy at integer time shown in Figure 5a corresponds to the outer face of the three dimensional diagram in Figure 5b (ε=0).

The predictable and unpredictable information components defined by Equations ((9)–(11)) can be calculated for the fractional transition matrix Equation (17), for any value of the fractional order parameter 0<ε≤1. In the present section, without loss of generality, we discuss the case of symmetric chain, q=p. For a symmetric chain, the densities of both states are equal, $\pi =\left[\frac{1}{2},\frac{1}{2}\right]$, so that $H\left(p,p\right)\equiv H\left(p\right)=-\log_{2}\frac{1}{2}=1$ bit, uniformly for all 0<p<1 (Figure 5a). The excess entropy Equation (9) quantifying predictable information encoded in the historical sequence of showed sides for a symmetric chain reads as follows [38]:

(22) $$E\left(p,p\right)\equiv E\left(p\right)=1-H\left(S_{t}|S_{t-1}\right)=-p\cdot \log_{2}p-\left(1-p\right)\log_{2}\left(1-p\right).$$

Forecasting the future state through discovering patterns in sequences of shown sides Equation (22) loses any predictive power when the coin is fair, $p=\frac{1}{2}$, but $E\left(p\right)=1$ bit when the series is stationary (i.e., p=0, or p=1). The mutual information Equation (10) measuring the reliability of the guess about the future state provided the present state is known [38],

(23) $$\begin{array}{ccccc} G\left(p\right) & = & p\cdot \log_{2}p & + & \left(1-p\right)\cdot \log_{2}\left(1-p\right)-2p\left(1-p\right)\cdot \log_{2}2p\left(1-p\right)\\ & & & - & \left(p^{2}+{\left(1-p\right)}^{2}\right)\cdot \log_{2}\left(p^{2}+{\left(1-p\right)}^{2}\right), \end{array}$$

increases as p≳0 (p≲1) attaining maximum at p≈0.121 (p≈0.879). The effect of destructive interference between two incompatible hypotheses about alternating the current state (p≳0) and repeating the current state (p≲1) culminates in fading this information component when the coin is fair, p=1/2 (Figure 5a). The difference between the entropy rate $H\left(S_{t}|S_{t-1}\right)$ and the mutual information $G\left(p\right)$ may be viewed as the “*degree of fairness*” of the coin that attains maximum ($U\left(p\right)=1$ bit) for the fair coin p=1/2 (see Figure 5a).

The entropy decomposition presented in Figure 5a for “integer”-order flipping (ε=0) evolves over the fractional order parameter, 0<ε≤1 as shown in Figure 5b: the decomposition of entropy shown in Figure 5a corresponds to the outer face of the three dimensional Figure 5b. When p=1, the sequence of coin sides shown in integer flipping is stationary, so that there is no uncertainty about the coin tossing outcome. However, the amount of uncertainty for p=1 grows to 1 bit, for fractional flipping as ε→1. When ε=1, the repetition probability of coin sides equals its relative frequency, $p_{1}=\pi_{1}=1/2$, and therefore uncertainty about the future state of the chain cannot be reduced anyway, $H\left(1/2\right)=U\left(1/2\right)=1$ bit. Interestingly, there is some gain of predictable information component G(p) for p=1 as ε≲1 (see Figure 5b). The information component G(p) quantifies the goodness of guess of the flipping outcome from the present state of the chain, so that the gain observed in Figure 5b might be interpreted as the reduction of uncertainty in a stationary sequence due to the choice of the present state, “0” or “1”. Despite the dramatic demise of unpredictable information for fractional flipping as ε→0, the fair coin (p=1/2) always stays fair.

## 6. Conclusions

A simple Markov chain generating binary sequences provides us with an analytically computable and telling example for studying conditional information quantities that quantify predictable and unpredictable information about the future states of the chain. The destructive interference between the mutually incompatible hypotheses about the forthcoming state of the chain results in damping of predictable information for a completely unpredictable, fair coin.

We have introduced and studied the fractional order generalization of the Markov chain defined as a convergent binomial series in the “integer”-order transition matrix. The proposed concept of fractional order Markov chain (fractional coin flipping) is similar to fractional random walks [8,9,10,11] and to the fractional order signal processing techniques generalizing the conventional integer order models—autoregressive integral moving average [14,15]. The backward-shift fractional order transition operator averages over all future states of the “integer”—order Markov chain exhibiting properties of long-time dependence, including the entanglement of state repetition probabilities assumed to be the independent parameters of the “integer”-order model.
